# Preliminary Study: Environmental Assessment of Perchloroethylene in Dry-Cleaning Facilities in the UAE

**DOI:** 10.1155/2018/1732906

**Published:** 2018-08-14

**Authors:** Sedra Habib, Hafiz O. Ahmed, Naema Al-Muhairi, Reem Ziad

**Affiliations:** Department of Environmental Health, College of Health Sciences, University of Sharjah, Sharjah, UAE

## Abstract

**Background:**

Perchloroethylene (PERC) is a widely spread cleaning solvent, used in nearly all dry-cleaning facilities. It has been declared as “probable human carcinogen” by the International Agency for Research on Cancer (IARC) due to its hazardous and toxic effects on human health. The study aimed at assessing the exposure of PERC among dry-cleaning workers at four different dry-cleaning facilities in the UAE.

**Methods:**

The four dry-cleaning facilities, using PERC in one of the cities of the UAE, were selected. Draeger perchloroethylene 10/b detector tubes along with a Draeger accuro pump were used to estimate the levels of PERC exposure in three main selected positions in each of the facilities.

**Results:**

The results showed that the second selected position had the highest amounts of PERC exposure above the international and local standards in 3 out of 4 selected facilities. The workers at position 2, who were not using any of the provided personal protective equipment, were at the highest risk of developing PERC-related health problems.

**Conclusion:**

It is important to install local exhaust ventilation systems and monitoring devices of PERC concentrations in these facilities, along with raising the awareness of workers about the health effects of PERC and the importance of using personal protective equipment (PPE) while performing their job.

## 1. Introduction

Perchloroethylene is a clear, colorless, and nonflammable synthetic liquid chemical that is volatile and readily evaporates at room temperature [1]. Perchloroethylene is also known as tetrachloroethylene and tetrachloroethene and abbreviated as PERC. It has been used as an intermediate in the synthesis of fluorocarbons, an insulating/cooling fluid in electric transformers, and as a veterinary medication against worms [2]. It is widely known for its use in dry cleaning of fabrics and for metal-degreasing operations, as it accounts for 80% to 85% of all dry-cleaning fluids used [3].

Short-term inhalation of tetrachloroethylene at very high levels can lead to irritation of the nose and throat and depress the central nervous system. Central nervous system, liver, kidneys, respiratory system, eyes, and skin are targeted organs for PERC toxicity in humans [4]. The neurobehavioral function of healthy individuals is affected adversely as a result of chronic, environmental exposure to PERC. The persistent solvent induced visual contrast sensitivity defects which increase the risk of developing neurological diseases. Chronic inhalation exposure to PERC is associated with headaches, impaired cognitive, motor neurobehavioral functioning, color vision impairment [5], cardiac arrhythmia, liver damage, and adverse effects on the kidneys [6]. There is also some evidence of an association between PERC and increased risk of certain cancers in dry-cleaning workers exposed for many years [7]. National Institute for Occupational Safety and Health (NIOSH) has declared PERC as potential occupational carcinogen. National Toxicology Program has designated it as reasonably anticipated to be a human carcinogen, whereas International Agency for Research on Cancer (IARC) has designated PERC as a probable human carcinogen [8].

Occupational exposure to perchloroethylene occurs primarily through inhalation and dermal contact at workplaces, where perchloroethylene is produced or used [9]. Therefore, this preliminary study has aimed to assess the exposure of perchloroethylene among dry-cleaning workers at four different dry-cleaning facilities in the UAE.

## 2. Materials and Methods

### 2.1. Sample Selection

The four dry-cleaning facilities, using PERC in one of the cities of the UAE, have been selected. Each facility worked for 8 hours per day with approximately three dry-cleaning loads.

### 2.2. Description of the Facilities' Process

Dry cleaning is any cleaning process for clothing and textiles using a chemical solvent other than water, usually perchloroethylene. Dry cleaning can be done using two main systems: opened transfer machines system and closed-loop dry-to-dry systems. The open dry-cleaning systems are the old type and damage the environment; therefore, they have been replaced with the closed systems in almost all facilities in the UAE.

In closed-loop dry-to-dry systems, clothes and fabrics are first inspected by professional dry cleaners, who are responsible for treating all major stains independently before the garment is loaded into the dry-cleaning machine. This step enables the elimination of all major stains during the actual cleaning process. It is then placed in the machine that has a filled solvent, taken from big perchloroethylene drums (200 L capacity). This procedure is carried out using installed mechanical pipes or manually using hoses to extract the solvent into the machine. The machine rotates swishing clothes around in the liquid that loosens the stain.

The dirt either ends up in a filter or is separated from the solvent through the process of distillation. PERC is sealed up in the machine and reused with each wash that is transformed to vapor from liquid and back again. The clothes are then taken out of the machine to a body steam press, which uses steam to evaporate the remains of perchloroethylene and its strong odor on the fabrics. It also helps in removing major wrinkles before it is ironed.

### 2.3. Measurement of PERC

The workers are mainly exposed to perchloroethylene, while working in three main working positions:

- Position 1: extracting PERC solvent from the drum to fill the dry-cleaning machine
- Position 2: unloading the clothes from the dry-cleaning machine into the tray
- Position 3: preparing clothes for steam pressing.

### 2.4. Procedure

**Figure 1:**
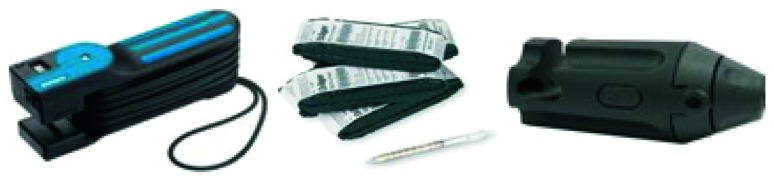
Device measuring PERC.

The Draeger perchloroethylene 10/b (CH 30701) detector tubes out of a variety of assessment methods of perchloroethylene were used with a Draeger accuro pump to estimate the levels of PERC exposure in each of the facilities (Figure 1). These tubes contained an inert carrier impregnated with a reagent. The ends of the tube were broken, and one side of the tube was connected to the hand operated dreamer pump. As the pump was pressed, ambient air was pulled through the tube and the perchloroethylene reacted with the reagent. This reaction changed the color of the tube from grey to orange, which indicated the concentration of PERC as shown in the following reaction:

(1) $$\begin{array}{c} {\text{CCl}}_{2}={\text{CCl}}_{2}+{\text{MnO}}_{4}⟶{\text{Cl}}_{2}\\ {\text{Cl}}_{2}+\text{o}-\text{toluidine}⟶\text{orange}\;\;\mathrm{reaction}\;\;\mathrm{product} \end{array}$$

### 2.5. Calculations

The actual concentrations were calculated after taking measurements from the selected positions, using the following equation:

(2) $$\begin{array}{c} \text{Concentration}\left(\text{in}\;\;\mathrm{ppm}\right)=\frac{3*x\;\;\mathrm{reading}\;\;}{\text{number}\;\;\mathrm{of}\;\;\mathrm{strokes}}*\text{ theoritical}\;\;\mathrm{strokes}. \end{array}$$

The concentrations of PERC were compared with the following standards, since the average exposure time of the workers to PERC during the whole work shift at each position was about 15 minutes:

- The American Conference of Governmental Industrial Hygienists (ACGIH) short-term exposure limit (STEL) which is 100 ppm for 15 minutes.
- The Occupational Safety and Health Administration (OSHA) Ceiling defined as concentration that should not exceeded 200 ppm for 5 minutes in any 3-hour period, with a maximum peak of 300 ppm.
- The United Arab Emirates (UAE) STEL, which is 100 ppm for 15 min.

## 3. Results

### 3.1. Dry-Cleaning Facility A

Table 1 shows the dry-cleaning facility A, which is one of the largest dry-cleaning facilities in the city located in the industrial area. The concentration of the first position (7.71–9 ppm) was found to be much lower than these standards. This is because the opening of the PERC drum is usually very small at this position from which the solvent is extracted. It only allows for a hose or a tube to be inserted in, which does not allow the volatile PERC to be emitted at high concentrations.

Table 2 shows that the minimum and maximum concentrations of PERC in position 2 exceeded the ACGIH STEL and UAE STEL Standards. It also shows that the maximum concentration was equal to OSHA Ceiling, which possesses a great health risk to the workers. Fabrics at this position which are unloaded from the dry-cleaning machine into the tray still have large amounts of PERC adhered to them. The clothes that are unloaded from the dry-cleaning machine into the tray still have large amounts of PERC adhered to them. These amounts of PERC have not been evaporated, and at this position, the worker is very close to the clothes and fabrics than any other position while performing the task.

The concentrations of PERC at position 3 were very low, ranging from 10 to 18 ppm. It was found to be below all the standards because PERC is a highly volatile solvent. Most of the PERC that remained adhered on the clothes after dry cleaning were emptied from the machine till the point they are prepared for pressing. This process takes around 5 minutes, which is enough for PERC to evaporate and leaving small concentrations at this position. However, the general ventilation system and air conditioning observed at this dry-cleaning facility were in good conditions. It is important to note that workers were not using any kind of personal protective equipment (PPE), when dealing with PERC during working hours, which increases their risk of developing adverse health effects.

### 3.2. Dry-Cleaning Facility B

The concentrations of PERC in dry-cleaning facility B were relatively low as compared to the facility A (Table 1). None of the concentrations measured at three different positions in this facility was found to be above ACGIH STEL, UAE STEL, and OSHA Ceiling Standards (Table 2). Therefore, the workers in this facility were not exposed to any high concentration that might pose a threat to their health during their work shifts. The general ventilation system was well maintained, but no local exhaust ventilation system was observed. However, none of the workers was using any kind of PPE except normal plastic gloves, which is not effective for this type of chemical. Moreover, it will not protect the user from the chemical, if it comes in direct contact with skin.

### 3.3. Dry-Cleaning Facility C

The third dry-cleaning facility C is a relatively small facility located in the residential building. Results of the concentrations have been listed in Table 1, which has shown that the first position had very low concentrations (4–10 ppm). Positions 1 and 3 had relatively low concentrations that were below ACGIH STEL (100 ppm), OSHA Ceiling (200 ppm), and UAE STEL (100 ppm) Standards (Table 2). However, when comparing the second position to these standards, it was extremely high (510 ppm) and exceeded all the standards. Therefore, workers at this position are exposed to extremely high amounts of PERC, which possesses a great risk to develop adverse health effects in workers within a short period of time. Such high concentration is explainable because in this particular facility and at position 2, the worker did not wait for the machine to take its full cycle to finish the dry-cleaning process. This shortens the time of the dry-cleaning load in order to accelerate the pace of the work and finish large amounts of loads in a shorter period of time. This is one of the very bad practices done in dry-cleaning facilities, which is extremely dangerous as it leaves much greater concentrations of PERC adhered to the clothes. PERC is not extracted before unloading, which results in extremely high concentration of PERC to which the worker is exposed. In this facility, workers were not using any kind of PPE when performing their job, except a normal dust mask. However, the dust mask does not protect the workers from this harmful chemical.

### 3.4. Dry-Cleaning Facility D

The dry-cleaning facility D is also a small facility located in one of the residential buildings. In this facility, both local and general ventilation systems are adopted and strong AC systems are located at different areas. Moreover, it also provides good hygienic practices, regular maintenance checkups, and the guidelines of Dry Clean and Laundry Institute (DLI). The concentrations of PERC in this facility at the first and third positions were almost insignificant and below all the standards. However, the maximum concentration at the second position (240 ppm) exceeded the standards. This might be due to the bad practices done in dry-cleaning facilities mentioned previously. As in other facilities, workers used normal plastic gloves which are not appropriate for this chemical.

## 4. Discussion

The findings of the present study have demonstrated that the workers exposed to PERC are at risk of developing acute or chronic adverse health effects, depending on the concentrations of PERC. Draeger detector tubes (instantaneous measuring device) have been used, which provide an assessment of PERC concentration within a time interval of a few seconds to a few minutes. It appears to be adequate as a screening device to measure PERC exposures of workers in the dry-cleaning industry. Earnest et al. [10] carried out a study to determine the concentrations of perchloroethylene in commercial dry cleaners at Teasdale Fenton Cleaners Cincinnati, Ohio, using Draeger® perchloroethylene detector tubes. The authors reported that the concentration ranged from 2 to 75 ppm with peak concentration over 300 ppm during the transfer operation and this is consistent with the finding of this study that the concentration of PERC was high in the second position (when unloading the clothes from the dry-cleaning machine into the tray).

A study conducted by McKernan et al. investigated the feasibility of conducting biological exposure to PERC among the workers at dry-cleaning sectors. The results showed that the concentration of PERC in blood has been used as a biological index to monitor. However, it is field sensitive but is an appropriate surrogate [6].

The results concluded that the second selected position (when unloading the clothes from the dry-cleaning machine into the tray) , in 3 out of 4 selected facilities, had the highest amounts of PERC exposure to workers. Therefore, this position had the greatest health risk on the workers. All the measured positions were found to be below the standards for dry-cleaning facility B. The concentrations in positions 1 and 3 were low and did not exceed any standards. At position 1, PERC drums are usually located outside the facility in open air, and the opening of the PERC drum is usually very small. It only allows for a hose or a tube to be inserted in and does not allow the volatile PERC to be emitted to workers at high concentrations. The concentrations were low at position 3 because the clothes were emptied from the dry-cleaning machine and left outside for a couple of minutes. It allowed the remaining adhered PERC to evaporate before preparing them for pressing; therefore, only small amounts of PERC were detected at this position. General ventilation systems provided in all facilities were not enough and needed to be enhanced. Majority of the facilities only had general ventilation system. There was no local exhaust ventilation system installed to eliminate PERC concentration from the source point, except for dry-cleaning facility D. Therefore, it is necessary to provide this type of ventilation system to enhance working conditions and reduce exposure. None of the facilities had monitoring devices to check for PERC levels. If massive concentrations were volatilized in the facility, monitors would give an alert; therefore, it is recommended to install this specific type of monitoring devices in all facilities. Improper storage at some facilities must be well maintained according to rules and regulations specified in the PERC Material Safety Data Sheet; otherwise, it leads to high amounts of exposure. Moreover, proper maintenance and leak detectors are important ways to avoid leakage, which reduces exposure to PERC.

## Figures and Tables

**Table 1:** Concentration of PERC (in ppm) (min-max) at each dry-cleaning facility.

Dry-cleaning facility	Position 1	Position 2	Position 3
A	7.71–9	150–200	10–18
B	10–75	51–63	7–8.4
C	4–10	60–510	3–8.4
D	2.72–3.38	60–240	1.15–2.33

**Table 2:** Comparison of the concentration of PERC (in ppm) from each dry-cleaning facility with the International Standards and UAE Standards.

Dry-cleaning facility A	ACGIH STEL: 100 ppm (as a 15 min TWA)	OSHA ceiling: 200 ppm (for 5 min in any 3 h period).	UAE Standards (AD EHSMS RF STEL): 100 ppm.
Position 1: 7.71–9	Below limit	Below limit	Below limit
Position 2: 150–200	Above limit	Max. concentration on boundaries	Above limit
Position 3: 10–18	Below limit	Below limit	Below limit

Dry-cleaning facility B	ACGIH STEL: 100 ppm (as a 15 min TWA)	OSHA ceiling: 200 ppm (for 5 min in any 3 h period)	AD EHSMS RF STEL: 100 ppm
Position 1: 10–75	Below limit	Below limit	Below limit
Position 2: 51–63	Below limit	Below limit	Below limit
Position 3: 7–8.4	Below limit	Below limit	Below limit

Dry-cleaning facility C	ACGIH STEL: 100 ppm (as a 15 min TWA)	OSHA ceiling: 200 ppm (for 5 min in any 3 h period)	AD EHSMS RF STEL: 100 ppm
Position 1: 4–10	Below limit	Below limit	Below limit
Position 2: 60–510	Max. concentration above limit	Max. concentration above limit	Max. concentration above limit
Position 3: 3–8.4	Below limit	Below limit	Below limit

Dry-cleaning facility D	ACGIH STEL: 100 ppm (as a 15 min TWA)	OSHA ceiling: 200 ppm (for 5 min in any 3 h period)	AD EHSMS RF STEL: 100 ppm.
Position 1: 2.72–3.38	Below limit	Below limit	Below limit
Position 2: 60–240	Max. concentration above limit	Max. concentration above limit	Max. concentration above limit
Position 3: 1.15–2.33	Below limit	Below limit	Below limit

## Data Availability

The data (results of the measurements of perchloroethylene) used to support the findings of this study are included within the article.
